# Depression in older breast cancer survivors

**DOI:** 10.1186/1471-2482-12-S1-S14

**Published:** 2012-11-15

**Authors:** Paola Frazzetto, Marco Vacante, Michele Malaguarnera, Ernesto Vinci, Francesca Catalano, Emanuela Cataudella, Filippo Drago, Giulia Malaguarnera, Francesco Basile, Antonio Biondi

**Affiliations:** 1 Department of Senescence, Urological and Neurological Sciences, Cannizzaro Hospital Via Messina 829, 95125, University of Catania, Italy; 2 International PhD programme in Neuropharmacology, University of Catania, Italy; 3 Department of Oncology, Ospedale di Enna, Italy; 4 Breast Unit, Department of Oncology, Cannizzaro Hospital Via Messina 829, 95125, Catania, Italy; 5 Department of General Surgery, Section of General Surgery and Oncology, Vittorio Emanuele Hospital, Via Plebiscito 628 University of Catania, 95123 Catania, Italy

## Abstract

**Background:**

Breast cancer is the most commonly diagnosed cancer among U.S. women .The 5-year survival rate for this tumour is nowadays 85%, and the 61% of these women are still alive at 15 years. When depression symptoms are present as a consequence of breast cancer treatments, they may interfere negatively with patients’ quality of life. The aim of this study was to examine the effects of breast cancer treatment on the quality of life and the impact of depression on the health-related life.

**Methods:**

We enrolled 173 women aged 65-75 years with early stage breast cancer diagnosed over the last 10 years, initially recruited to participate in a study examining heath-related quality of life in the first 5 years after breast cancer diagnosis. Participants were divided into four groups: 1) 46 breast cancer survivors (aged 65-70); 2) 62 women diagnosed with breast cancer (aged 65-69); 3) 32 women with recurrent breast cancer after 10 years (aged 66-75); 4) 30 women in good health status (aged 60-70). The Geriatric Depression Scale was used as a routine part of a comprehensive geriatric assessment. Collection of data for the application of instruments, such as sociodemographic variables (age, educational level, social state) and clinical date (stage and time of the disease and treatment), was carried out by trained researcher assistants.

**Results:**

Our results demonstrated the correlation between depression and previous cancer experiences. In fact, in patients with cancer experience, the grade of depression was significantly higher compared to healthy subjects. Furthermore, we demonstrated that the patients with recurrent breast cancer were severely depressed compared to other groups.

**Conclusions:**

A high percentage of participants were identified as having emotional and/or well being problems. Further investigations on the cause of depression problems cancer-related are needed.

## Background

Breast cancer is the most commonly diagnosed cancer among U.S. women [1]. The mean age of all women diagnosed with breast cancer is 61 years, and women aged 75-79 years have the highest incidence of the disease [2]. Most encouraging fact is represented by the topic that women with a history of breast cancer are the largest group of female cancer survivors. In fact, the 5-year survival rate is nowadays 85% and 61% of these women are still alive at 15 years. Approximately 4.4 million women who were diagnosed with breast cancer in the last 5 year currently are alive, making breast cancer the single most prevalent cancer in the world [3]. The term of “breast cancer survivors” is here used to refer to women with a history of breast cancer and to emphasize the issues concerning with quality of life in older women disease-free breast cancer. So, the main issue is the correct understanding of the variables that influence quality of life (QOL) among long-term survivors of breast cancer. Effective treatments for breast cancer can produce a life expectancy of 10 or more years, increasing concern for patients’ QOL [4-7]. When depression symptoms are present as a consequence of breast cancer treatments, they may interfere negatively with patients’ QOL, above all on QOL of long-term survivors. In addition, when a woman develops breast cancer all family members may develop some sort of illness [8]. It is recognized that breast cancer diagnosis and its treatment may have a significant practical and emotional impact on the entire family, but particularly on partners [9]. The aim of this study was to examine the effects of breast cancer treatment on the quality of life and the impact of depression on the health-related life.

## Methods

All eligible patients to participate in this study were older women with early stage breast cancer diagnosed over the last 10 years (Table 1). Participants were initially recruited to participate in a study examining heath-related quality of life in the first 5 years after breast cancer diagnosis. The study population was composed of 173 women aged 65-75 years who have been recruited into the department of geriatrics at Cannizzaro Hospital, University of Catania. Participants were divided into four groups: 1) 46 breast cancer survivors (aged 65-70); 2) 62 women diagnosed with breast cancer (aged 65-69); 3) 32 women with recurrent breast cancer after 10 years (aged 66-75); 4) 30 women in good health status (aged 60-70). Data for the analyses were obtained from 1) hospital medical records abstracted for the hospitalization at the most definitive treatment of breast cancer, 2) a follow-up survey. The initial consent form signed by all of the subjects included a statement of willingness to be contacted about future studies. The Geriatric Depression Scale (GDS) was used as a routine part of a comprehensive geriatric assessment. The GDS is a measure that is designed to assess depression in older adults and was developed and standardized strictly on samples of older people. The scale consists of a 100 questions about which selected 30 item self-report assessments designed specifically to identify depression in elderly. The GDS questions are answered “yes” or “no”, which is thought to be simpler than scales that use a five-category response set. One point is assigned to each answer and the cumulative score is rated on a scoring grid. The grid sets a range of 0-9 as “normal”, 10-19 as “middle depressed”, and 20-30 as “severely depressed”. A score of 10 or 11 or lower is the usual threshold to separate depressed from non-depressed patients. Collection of data for the application of instruments, such as sociodemographic variables (age, educational level, social state) and clinical date (stage and time of the disease and treatment), was carried out by trained researcher assistants. The instruments were submitted directly by the patients.

**Table 1:** Demographic characteristics of subjects included in the study.

	Breast Cancer Survivors	Breast Cancer	Recurrent breast cancer	Healthy subjects
**Number**	46	62	30	35

**Age (years)**	69.5 ± 4.7	67.5±5.2	69.8±5.2	68.2±4.2

**FC (bpm)**	78±3	65±3	80±4	70±4

**PAS (mmHg)**	138±10	145±8	142±10	144±15

**PAD (mmHg)**	82±8	85±9	86±6	87±3

## Results and discussion

Our results demonstrated the correlation between depression and previous cancer experiences. In fact, in patients with cancer experience, the grade of depression was significantly higher compared to healthy subjects. Furthermore, we demonstrated that the patients with recurrent breast cancer were severely depressed compared to the other study groups. In our study, subjects with breast cancer and breast cancer survivor showed mild depression levels. As regards the GDS score, the comparison of breast cancer survivors and subjects with breast cancer showed significant group differences (Table 2). This study evaluated the prevalence of depression symptoms in older breast cancer survivors. Cancer survivors lose the meaning of health and life itself following a diagnosis of cancer. Depression, anxiety, fatigue and sleep disturbance are among the most commonly reported problems experienced by cancer survivors, above all changes in physical form and psychosocial modifications. There are several complications of cancer and its treatment that can cause or exacerbate an alteration in mood, for example uncontrolled pain that can cause anxiety, despair of relief of their suffering, and at times suicide. In addition, changes in physical form due to the cancer or its treatment, such as mastectomy, could have a large impact on the psyche and contribute to depression mood. Finally, changes in the life of the person with cancer like being away from work or not being able to do the usual roles at home may have affect the person’s self-esteem. As well, the spiritual side of a person may be severely affected by their illness. Family members usually provide personal care and emotional support for the duration of the cancer experience. Caregivers and family members often require, but do not receive, the respite, health care, psychosocial, and financial assistance they need in meeting the many needs of cancer survivors in their lives. In our study, we demonstrated that the prevalence of depression was higher among breast cancer survivors than in breast cancer patients.

**Table 2:** Percentage of subjects in each group according to the GDS score.

	Breast Cancer survivors	Breast cancer	Recurrent breast cancer	Healthy subjects
**Normal**	21,7%	51,6%	16,6%	71,42%

**Middle depressed**	43,4%	32,2%	33,3%	20%

**Severely depressed**	34,7%	24,19%	50%	8,57%

GDS score: normal 0-9, mild depression 10-19, severe depression 20-30.

## Conclusions

A high percentage of participants were identified as having emotional and/or well being problems. Moreover the participants had a physical activities level that was insufficient to yield expected benefits. The focus of further researches should be the investigation of the cause of depression problems cancer-related and the need for a better understanding of why cancer survivors decrease their level of physical activities following a cancer diagnosis. Increasing physical activities to a level that is sufficient for cancer survivors to receive known benefits (for example, reduced depression, increased functional well being) should be the goal of any intervention.

## List of abbrevations used

QOL: Quality of Life; GDS: Geriatric Depression Scale

## Competing interests

The authors declare that they have no competing interests.

## Authors’ contributions

PF, MV, MM: conception and design, interpretation of data, drafting the manuscript, given final approval of the version to be published; EV, FC, EC, GM: acquisition of data, drafting the manuscript, given final approval of the version to be published; FD, FB, AB: critical revision, given final approval of the version to be published.
